# Mental health and psychosocial support in humanitarian settings: research priorities for 2021–30

**DOI:** 10.1016/S2214-109X(23)00128-6

**Published:** 2023-04-25

**Authors:** Wietse A Tol, PhuongThao D Le, Sarah L Harrison, Ananda Galappatti, Jeannie Annan, Florence K Baingana, Theresa S Betancourt, Cecile Bizouerne, Julian Eaton, Michelle Engels, Zeinab Hijazi, Rebecca R Horn, Mark J D Jordans, Brandon A Kohrt, Phiona Koyiet, Catherine Panter-Brick, Michael Pluess, Atif Rahman, Derrick Silove, Mark Tomlinson, José Miguel Uribe-Restrepo, Peter Ventevogel, Inka Weissbecker, Alastair Ager, Mark van Ommeren

**Affiliations:** a Section of Global Health, Department of Public Health, University of Copenhagen, Copenhagen, Denmark; b Peter C Alderman Program for Global Mental Health, HealthRight International, New York, NY, USA; c Athena Institute, Vrije Universiteit Amsterdam, Amsterdam, Netherlands; d School of Global Public Health, New York University, New York, NY, USA; e International Federation of Red Cross Red Crescent Societies Reference Centre for Psychosocial Support, Copenhagen, Denmark; f Mental Health and Psychosocial Support Network (MHPSS.net), Colombo, Sri Lanka; g Airbel Impact Lab, The International Rescue Committee, New York, NY, USA; h WHO African Region Advisor, MNS, Brazzaville, Congo; i Boston College, School of Social Work, Research Program on Children and Adversity, Chestnut Hill, MA, USA; j Mental Health, PsychoSocial Support and Protection Sector, Action Contre la Faim, Paris, France; k Centre for Global Mental Health, London School of Hygiene & Tropical Medicine, London, UK; l CBM Global, Amstelveen, Netherlands; m International Medical Corps, Washington, DC, USA; n Mental Health Unit, Programme Division, UNICEF, New York, NY, USA; o Institute for Global Health & Development, Queen Margaret University, Edinburgh, UK; p Centre for Global Mental Health, Institute of Psychology, Psychiatry and Neuroscience, King's College London, London, UK; q Amsterdam Institute of Social Science Research, University of Amsterdam, Amsterdam, Netherlands; r Department of Psychiatry and Behavioral Sciences, George Washington University, Washington, DC, USA; s World Vision International, Nairobi, Kenya; t Department of Anthropology and Jackson School of Global Affairs, Yale University, New Haven, CT, USA; u Department of Biological and Experimental Psychology, Queen Mary University of London, London, UK; v Institute of Population Health, University of Liverpool, Liverpool, UK; w University of New South Wales, Sydney, NSW, Australia; x Institute for Life Course Health Research, Department of Global Health, Stellenbosch University, Cape Town, South Africa; y School of Nursing and Midwifery, Queen's University Belfast, Belfast, UK; z Department of Psychiatry and Mental Health, Pontificia Universidad Javeriana, Bogotá, Colombia; aa Public Health Section, Division of Resilience and Solutions, United Nations High Commissioner for Refugees, Geneva, Switzerland; ab Department of Mental Health and Substance Use, World Health Organization, Geneva, Switzerland; ac Mailman School of Public Health, Columbia University, New York, NY, USA

## Abstract

We describe an effort to develop a consensus-based research agenda for mental health and psychosocial support (MHPSS) interventions in humanitarian settings for 2021–30. By engaging a broad group of stakeholders, we generated research questions through a qualitative study (in Indonesia, Lebanon, and Uganda; n=101), consultations led by humanitarian agencies (n=259), and an expert panel (n=227; 51% female participants and 49% male participants; 84% of participants based in low-income and middle-income countries). The expert panel selected and rated a final list of 20 research questions. After rating, the MHPSS research agenda favoured applied research questions (eg, regarding workforce strengthening and monitoring and evaluation practices). Compared with research priorities for the previous decade, there is a shift towards systems-oriented implementation research (eg, multisectoral integration and ensuring sustainability) rather than efficacy research. Answering these research questions selected and rated by the expert panel will require improved partnerships between researchers, practitioners, policy makers, and communities affected by humanitarian crises, and improved equity in funding for MHPSS research in low-income and middle-income countries.

## Introduction

Humanitarian crises, such as armed conflicts and disasters, have diverse effects on the mental health and psychosocial wellbeing of affected populations. To address these effects, mental health and psychosocial support (MHPSS) interventions have gained greater recognition throughout the past three decades.^1^ Improved knowledge is essential to support appropriate MHPSS responses in humanitarian crises. However, as in other areas of humanitarian work, important gaps remain between the focus and outputs of researchers, and the knowledge priorities of MHPSS practitioners and policy makers. In 2011, a consensus-based process to set MHPSS research priorities involved a wider group of stakeholders, including MHPSS researchers and practitioners.^2^, ^3^ In this Health Policy, we discuss the outcomes of a second, consensus-based, research prioritisation process, which sought to develop an updated MHPSS research agenda for the current decade, 2021–30.

## Methods

Our research prioritisation process had two interlinked goals. First, we were interested in the development of a renewed research agenda with input from a broad composition of current MHPSS stakeholders, with the knowledge that the MHPSS field has rapidly evolved in the past decade. Second, we wanted to understand how MHPSS research priorities might have shifted over time. Thus, instead of conducting a follow-up study, we generated a renewed consensus on MHPSS research priorities from the bottom up. To create this renewed consensus, we followed methods applied in the earlier MHPSS research prioritisation initiative^2^ and built on the methods used in similar initiatives in related global health fields.^4^, ^5^ In essence, these methods resemble adapted Delphi consensus-building techniques. Delphi processes usually engage experts in several rounds of structured consultation involving summary statements, ranking, and re-ranking to come to consensus.^6^ We engaged a larger group of people and a wider range of experts (including policy and practice experts) than in typical Delphi processes.

A key consideration was to incorporate both the voices of people implementing MHPSS programmes (ie, humanitarian workers) and people with lived experiences (ie, individuals affected by mental health and psychosocial effects of humanitarian crises) through a qualitative approach. We were also committed to strengthening collective ownership of the final research agenda by practitioners, policy makers, and researchers. In designing our approach, we therefore emphasised feasibility. In addition, the current initiative was implemented under the auspices of the Inter-Agency Standing Committee Reference Group for MHPSS in Emergencies, and was governed by a Funding and Policy Council and a Scientific and Practice Advisory Board (appendix pp 1–2). The initiative was implemented in three phases (figure).

**Figure:**
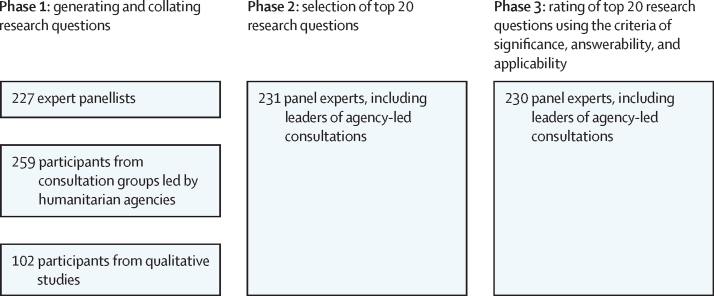
Overview of mental health and psychosocial support in humanitarian crises: setting consensus-based research priorities for 2021–30 (MHPSS-SET 2) study design

### Phase 1: generating and collating research options

We generated research questions through three sources: a panel of MHPSS experts, consultations led by humanitarian agencies, and a qualitative study. Expert panellists were consulted through emails and online surveys and included Scientific and Practice Advisory Board members and their nominations of other practitioners, researchers, and policy makers through several rounds of snowball sampling, including through Inter-Agency Standing Committee Technical Working Groups. We asked experts to nominate other members and aimed to ensure a panel with diverse experience and background with respect to gender, geographical location, and perspectives (appendix p 3). The consultations led by humanitarian agencies (a new addition since the 2011 priority-setting process) consisted of a resource package circulated through a social media campaign facilitated by MHPSS.net (by author AG), which was posted on their website, Facebook, Twitter, LinkedIn, and WhatsApp channels. The resource package consisted of a slide deck with instructions, a recorded instructional webinar (hosted on YouTube), and an online or email form to submit responses (appendix p 3). The qualitative study consisted of in-depth interviews and focus groups with people with lived experience, humanitarian workers, and policy makers in crisis-affected settings in Indonesia, Lebanon, and Uganda (appendix pp 4–5).

Participants across the three sources were asked to list their most important research questions in the field of mental health and psychosocial support in the next 10 years. Each expert panellist could list up to five research options (in line with the 2011 MHPSS research priority initiative), consultation groups led by humanitarian agencies could list up to ten questions (estimated to be feasible to cover in an in-person group format), and the qualitative study participants could list as many as they wanted (to increase the potential for saturation in themes from qualitative interviews).

We subsequently collated research options using qualitative thematic data analysis techniques, building on participants' words as much as possible. For feasibility reasons, our intention was to consolidate all generated options into a list of 100 maximum unique research options. We therefore grouped similar questions together and created subcategories (ie, dropdown options) under the more encompassing research question. In addition, we only included research questions that were mentioned by at least five participants. We grouped research options into overall themes and subthemes using top-down (with the five overall themes from 2011) and bottom-up in-vivo strategies (ie, creating new overall themes when necessary, and building subthemes with the terms and phrasing submitted by participants).

### Phase 2: selection of top 20 research options

The second phase included the panel of MHPSS experts, comprising the Scientific and Practice Advisory Board members and individuals who spearheaded the consultations led by humanitarian agencies in the first phase. Participants were sent a password-protected Qualtrics survey in which they could select their top 20 research questions. Options were presented by theme in random order. We analysed how often each research option was selected by participants to identify the 20 most prioritised research options. We asked MHPSS experts in the panel to self-identify if they were also people with lived experience (as defined in the previous section).

### Phase 3: rating of top 20 research options

We asked the same participants from phase 2 to rate the top 20 research options using pre-set criteria. The Scientific and Practice Advisory Board, through internal consensus over email, selected three criteria from the five that were previously selected in 2011: significance (does the question need answering), answerability (is a study to answer this question feasible), and applicability (will an answer to this question help to influence humanitarian policy and practice). Participants rated each criterion as being either essential or unimportant, and we then averaged the ratings across all three criteria and from all participants to rank the top 20 research options.

We had delays with implementing the process and therefore finalised results in 2022. In 2020 and 2021, initial findings were presented at the annual meetings for the Inter-Agency Standing Committee Reference Group for MHPSS, to invite feedback and stimulate continued ownership. This Health Policy presents our findings to an external audience for the first time.

Upon completion of the analysis, we reflected on differences with the 2011 MHPSS research priorities in meetings with the Funding and Policy Council and the Scientific and Practice Advisory Board. Differences were also discussed by email among the author team, made up of the implementation team leaders, chairs of the Funding and Policy Council and the Scientific and Practice Advisory Board, and board members interested in coauthoring (all board members were invited). For ease of reference, the 2011 MHPSS research priorities are provided in the appendix (pp 6–8).

## Results

A total of 588 participants generated 1503 research questions: 227 expert panellists generated 1046 questions, 259 participants from 21 consultation groups led by humanitarian agencies generated 179 questions, and 102 participants from 33 in-depth interviews and eight focus group discussions generated 278 questions (table 1). We consolidated the 1503 research questions into a list of 61 research questions, grouped into six themes: problem assessment and analysis (nine questions), intervention benefits (16 questions), research and information management (six questions), context (seven questions), implementation and organisation of MHPSS interventions (15 questions), and special topics (questions regarding COVID-19, pandemics, and digital technology; eight questions).

**Table 1:** Participants in phase 1

		**Participants, n**	**Research questions generated, n**
Expert panel*		227	1046
	Researchers in LMICs	51	..
	Researchers in HICs	29	..
	Practitioners in LMICs	89	..
	Practitioners in HICs	8	..
	Policy makers in LMICs	21	..
	Policy makers in HICs	4	..
	Unknown	25	..
Consultations led by humanitarian agencies		259	179
	Community-based and non-governmental organisations, social enterprises, and online networks	216	..
	National MHPSS coordination groups	34	..
	National research and scientific organisations	9	..
Qualitative study		102	278
	People with lived experience	34	..
	Humanitarian practitioners	64	..
	Policy makers	4	..
Total		588	1503

HICs=high-income countries. LMICs=low-income and middle-income countries. MHPSS=mental health and psychosocial support.

* 22% of expert panellists self-identified as having lived experience of mental health concerns and psychosocial impacts of humanitarian crises.

Subsequently, 231 participants (panel experts and leaders of agency-led consultations) selected the 20 most important research options from the consolidated list. The five most frequently selected questions were chosen by 50% or more of the participants. From the consolidated list of questions, participants most commonly selected questions from the themes of problem assessment and analysis (6/9), intervention benefits (8/16), and research and information management (3/6).

As the final step, 230 panellists scored the top 20 research questions (table 2). Overall, research options related to potential intervention benefits were ranked of high importance: eight of the 20 research questions pertained to this theme (questions 4, 7, 10, 11, 13–15, and 20). Options related to the theme of problem assessment and analysis were ranked less prominently (questions 8, 12, and 16–19).

**Table 2:** Top 20 prioritised research questions and scores for 2021–30

	**Theme**	**Significance**	**Answerability**	**Applicability**	**Average**
(1) How can we strengthen the MHPSS workforce in humanitarian settings?	Implementation and organisation	94%	87%	91%	91%
(2) What are the appropriate methods to assess the outcomes and effects (ie, short-term and long-term benefits) of MHPSS interventions and approaches?	Research and information management	90%	84%	82%	85%
(3) How can we effectively develop MHPSS monitoring, evaluation, and research systems in humanitarian settings?*	Research and information management	89%	80%	86%	85%
(4) What is the added value of integrating or mainstreaming MHPSS services into other sectors (eg, education, WASH, social protection) in humanitarian settings?	Benefits or the effectiveness of interventions	89%	78%	86%	84%
(5) How can we better develop supervision models and strategies to address MHPSS needs in humanitarian settings?	Implementation and organisation	85%	82%	84%	84%
(6) What are the effectiveness and best practices of remote or digital MHPSS interventions?	Special topics (ie, digital technology, COVID-19, and pandemics)	87%	82%	82%	83%
(7) What is the impact of MHPSS interventions in humanitarian settings?*	Benefits or the effectiveness of interventions	89%	76%	85%	83%
(8) How do mental health and psychosocial concerns influence social and economic functioning (eg, economic outcomes, family functioning, social relations)?	Problem analysis	89%	78%	82%	83%
(9) How can we develop and adapt tools that are culturally and cross-culturally valid?	Research and information management	90%	78%	81%	83%
(10) How can we ensure the sustainability of MHPSS services in various settings and sectors?	Benefits or the effectiveness of interventions	93%	71%	83%	82%
(11) What should be the minimum or essential set of MHPSS services in humanitarian settings?	Benefits or the effectiveness of interventions	82%	73%	85%	80%
(12) What are the major risk factors and protective factors of MHPSS issues in humanitarian settings?*	Problem analysis	81%	80%	77%	79%
(13) How can we develop effective, multisectoral, multilayered interventions in humanitarian settings?	Benefits or the effectiveness of interventions	87%	71%	79%	79%
(14) What are the comparatively most optimal (eg, effective, efficient, cost-effective, safe) MHPSS interventions or responses to address issues in humanitarian settings?*	Benefits or the effectiveness of interventions	86%	67%	83%	79%
(15) How can we ensure effective participation of key stakeholders in MHPSS programmes?*	Benefits or the effectiveness of interventions	81%	74%	78%	78%
(16) What is the current understanding and what are the gaps in knowledge about MHPSS issues in humanitarian settings?	Problem analysis	75%	79%	73%	76%
(17) What are the most important MHPSS problems in humanitarian settings?*	Problem analysis	76%	76%	72%	75%
(18) What are the correlates of resilience in humanitarian settings?	Problem analysis	80%	69%	73%	74%
(19) How are the consequences of traumatic experiences and adversity, including childhood adversity, transmitted across generations?	Problem analysis	81%	61%	67%	70%
(20) What is the relationship between MHPSS programmes and peacebuilding, and how can peacebuilding be effectively promoted in MHPSS programmes?	Benefits or the effectiveness of interventions	73%	60%	67%	67%

MHPSS=mental health and psychosocial support. WASH=water, sanitation, and hygiene.

* Scores indicate percentages of respondents endorsing the stated criteria (significance, answerability, and applicability).

Most questions scored lower on answerability than on applicability and significance. A few questions scored higher on significance but lower on answerability, such as questions 10 (sustainability), 13 (development of multisectoral, multilayered programmes), and 19 (intergenerational transmission of adversity across generations). Agreement was broad in the rankings of professional participant subgroups, with some minor differences. For example, policy makers scored questions focused on digital interventions, resilience, and comparative cost-effectiveness higher than did implementers and researchers. However, we did not identify major differences in rankings among the 22% of participants who self-identified as having lived experience.

### Changes since the 2011 priority-setting exercise

We compared the top 20 MHPSS research priorities for 2021–30 with those previously identified for 2011–20 (appendix p 6). Although comparison is limited by variations in methods, we note three key differences. First, there is an indication that research priorities are different now: four of the five top research priorities in 2011 (ie, stressors faced in humanitarian crises [priority 1], how to assess needs [priority 2], local perceptions on the effects of MHPSS [priority 3], and adaptations to MHPSS interventions [priority 5]) no longer feature in the current top 20.

Second, although research priorities have changed, the overall emphasis remains analogous: the current research agenda still favours applied research questions over more fundamental, basic questions. For example, the top three research questions in the current agenda focus on how to strengthen the MHPSS workforce and how to improve programmatic monitoring and evaluation practices. In 2011, examples of practice-focused research questions included appropriate indicators for monitoring and evaluation, and the extent to which MHPSS programming covered locally perceived needs.

Finally, we note a difference in focus in the intervention-based research priorities: namely, a shift from prioritising basic questions on the effectiveness of interventions (such as the effectiveness of preventive, family-based and school-based MHPSS interventions) to prioritising systems-related questions and implementation research. Evaluating the effect of interventions still ranks seventh, but new research priorities are focused on the integration of MHPSS with different humanitarian sectors (priority 4), the minimum required set of MHPSS interventions (priority 11), and developing multilayered systems of care (priority 13).

## Discussion

We developed a new consensus-based agenda for MHPSS research for the current decade (2021–30). In formulating this forward-looking agenda, we engaged with diverse stakeholders (researchers, practitioners, policy makers, and people with lived experience) in a multistep process of generating, consolidating, and rating prioritised research questions.

We highlight four main conclusions and associated recommendations. First, the current MHPSS research prioritisation exercise—like that reported in the 2011 consensus^2^—provides a practice-oriented research agenda, rather than one focused on more fundamental questions about how mental health and psychosocial wellbeing manifest and what predicts them in humanitarian settings. For example, highly prioritised research questions concern strengthening and supervising the MHPSS workforce, identifying and implementing culturally relevant monitoring and evaluation practices, identifying intervention benefits (including those of digital interventions), and the application of MHPSS interventions in sustainable, integrated, multisectoral, and multilayered systems. With this overall focus, we believe a key recommendation remains that collaboration between MHPSS researchers, practitioners, and policy makers needs to be strengthened. This recommendation requires stronger input from practitioners in research efforts than is currently provided, more frequent and meaningful dialogue between stakeholders, and the application of research approaches to improve humanitarian practice (eg, building on a researcher's experience to strengthen programme monitoring and evaluation practices). Such a process was applied in developing interagency guidance on monitoring and evaluation,^7^, ^8^ and could also be applied in other areas.

Second, within the overall emphasis on practice-oriented questions, there has been a shift in research priorities compared with those of 10 years ago. Specifically, research on mapping MHPSS problems is ceding ground to a stronger focus on implementation research. This shift in focus could reflect a maturation of the MHPSS research field, as indicated by a larger body of epidemiological research^9^ and an increase in published studies evaluating MHPSS interventions.^10^, ^11^, ^12^ Epidemiological questions that remain consensus-based research priorities concern risk factors and protective factors, correlates of resilience, and intergenerational transmission of adversity, rather than merely identifying prevalence rates of depression and post-traumatic stress disorder. We believe these reorganised research priorities indicate a need for interdisciplinary, transdisciplinary, and systems-oriented approaches to implementation science, which focus on how evidence-based MHPSS practices can be scaled and sustained with the integration of robust quality-improvement systems into routine delivery settings (ie, outside of controlled research settings). Advances in epidemiological research to better understand wellbeing and mental health in a socioecological context are also required.

Third, we highlight a potential mismatch between the rich evidence base in scholarly literature and the emphasis of the MHPSS research priorities in the 2021 agenda-setting exercise. Despite a decade of work to generate a more rigorous evidence base for MHPSS interventions, the seventh most highly prioritised question of the 2021 agenda concerns the effect of these interventions. Similarly, research focused on problem analysis remains a priority, despite being the focus of several peer-reviewed publications.^13^, ^14^ These topics could have been included in the current research agenda to raise complementary questions. For example, queries regarding the effect of MHPSS interventions might be referring to the understanding of their benefits beyond those found in controlled trials and beyond the reduction in psychological symptoms. Research questions in the current research agenda might pertain to the wider effect of MHPSS interventions or the effect of less-researched interventions.^10^ In addition to the possibilities of mismatches between the existing scholarly literature and the 2021 priorities, scholarly research is unlikely to reach many practitioners and policy makers.^15^ To resolve this issue, scholars should work with their Communications and Policy colleagues, and other strategists, to translate their scientific findings in ways that are compelling to decision makers. Research that occurs in partnership between scholars and practitioners is also important, such that practitioners can discuss and influence research objectives and partnerships between local communities, researchers, and practitioners to increase the relevance of study objectives.^16^

Finally, there are few questions related to prominent themes in current humanitarian discourse, such as MHPSS research questions linked to the COVID-19 pandemic or the climate emergency. Although options pertaining to these topics were generated during the initial phases of the process, they were not frequently selected for the top 20. However, the prioritised question on digital tools to support MHPSS interventions is likely to have been preferentially selected considering operational challenges resulting from the COVID-19 pandemic. We anticipate that questions related to climate change will probably be prioritised more highly in future priority-setting exercises for MHPSS research.^17^

We highlight three main study limitations. First, our methods might have favoured the formulation of generic and interpretable research questions, rather than novel and complex ones. We sought to make the process of formulating and ranking research options as efficient as possible, to help engage busy humanitarian practitioners. This approach led to several pragmatic choices, such as limiting scoring criteria to three, confining options to a shortlist of the top 20 questions, and using super-ordinate questions with options for further specificity of lower-level topics. Although we hope these choices increased a willingness to respond, we possibly chose breadth of participants coverage over depth of questions.

Second, only the expert panel was consulted for all three surveys. However, the size and composition of the expert panel differed between the surveys, due to the progressive snowball sampling methods used and the availability of the panel members during each survey period. Across the project's duration, a total of 304 panel members participated in at least one survey, and 144 people participated in all three surveys. Additionally, the panel's expertise might have been heavily drawn from their experience in countries affected by humanitarian crises arising from political conflicts (appendix p 9), and panellists had to have a working knowledge of the English language. Finally, we found that the online format was difficult to implement with policy makers, limiting their inclusion in the sample of respondents to 6·5%. A face-to-face approach, such as the one we adopted with the qualitative study component (ie, direct interviews or focus group discussions) might have been more successful for this type of respondent.

In addition to discussing limitations inherent to the applied methods in this initiative, reflecting more broadly on the implementation and potential effect of setting consensus-based research priorities for the MHPSS field is important. For the current consensus-based research priorities to affect (and ultimately improve) MHPSS research practice, we believe two main challenges exist. First, answering the prioritised research questions will require funding. Exact figures are challenging to obtain, but a larger share of funding for humanitarian health research is from research donors who do not consider themselves to be part of the humanitarian system (eg, substantial funding from programmes such as Horizon 2020). Such funders might not be closely familiar with these research priorities, or be responsive to research, practitioner, and affected community priorities without targeted efforts. In addition, a notable but smaller proportion of humanitarian health research comes from humanitarian donors who are largely from Germany, the USA, and the UK.^18^ This funding situation requires crucial reflection, given that the largest populations affected by humanitarian crises live in low-income and middle-income countries (LMICs), and there are systematic inequities in global health research funding between high-income countries and LMICs. In line with recommendations concerning localisation in the humanitarian field^19^ and decolonisation in global health research,^20^ MHPSS research funding would benefit from: more direct funding for researchers from LMICs, so as to build knowledge infrastructures; an equitable voice for these researchers in LMICs in funding priorities and decisions from donors based in high-income countries; and—where possible—increased funding from LMICs.^21^, ^22^, ^23^

The second issue is that the research agenda will require uptake by diverse stakeholders in the MHPSS field. Unfortunately, research might not always reach policy and practice stakeholders.^24^ This situation could be resolved by strengthening research–practitioner partnerships (across all stages of research projects, and beyond completion), understanding how humanitarian policy makers and practitioners prefer research dissemination to be formatted and timed (and recognising the gap between researchers and decision makers as a complex system),^25^ and by improving researchers' dissemination skills.^24^, ^26^ In addition, stronger engagement of communities affected by humanitarian crises in MHPSS research would enhance the effect of the prioritised research questions.^27^

In conclusion, we see clear research priorities for a practice-based 2021–30 MHPSS agenda, which calls for a shift towards interdisciplinary and transdisciplinary implementation research to strengthen the integration of MHPSS interventions into scalable and sustainable delivery platforms. This target can be met through research on how to develop and support capacity and supervision in the global MHPSS workforce across sectors, how to ensure appropriate monitoring and evaluation of MHPSS interventions within programme implementation, and how to effectively scale and sustain the quality of evidence-based MHPSS interventions across diverse humanitarian sectors. Answers to the research questions prioritised in this consensus-based agenda require stronger linkages between MHPSS researchers, practitioners, and policy makers than is currently the case, in partnership with crisis-affected communities.

## Declaration of interests

We declare no competing interests.
