# Supplementary material for: Mental health and psychosocial support in humanitarian settings: research priorities for 2021–30
Source: Lancet Glob Health. 2023 Apr 25;11(6):e969–75. doi: 10.1016/S2214-109X(23)00128-6 (PMC10188364; doi:10.1016/S2214-109X(23)00128-6)
Supplement: Supplementary appendix [file mmc1.pdf]

THE LANCET
Global Health

Supplementary appendix

This appendix formed part of the original submission and has been peer reviewed. We post it as supplied by the authors.

Supplement to: Tol WA, Le PTD, Harrison SL, et al. Mental health and psychosocial support in humanitarian settings: research priorities for 2021–30. *Lancet Glob Health* 2023; published online April 25. https://doi.org/10.1016/S2214-109X(23)00128-6.

Appendix #1. Governance structure of MHPSS-SET 2: Membership of Funding & Policy Council (FPC) and Scientific & Practice Advisory Board (SPAB)

**MHPSS-SET 2 Funding and Policy Council (FPC)**		
**Ager,** Alastair<br>*Co-Chair*	As part of the study: Deputy Chief Scientific Adviser, DFID<br>Now: National Institute for Health Research, Queen Margaret University & Columbia University	UK, USA
**van Ommeren,** Mark<br>*Co-Chair*	Head, Mental Health Unit, MSD, World Health Organization	Switzerland
**Anand**, Nalini	Director, Center for Global Health Studies, Fogarty International Center, National Institutes of Health	USA
**Bardikoff**, Nicole	Program Officer, Grand Challenges Canada	Canada
**Baessler**, Judith	Head of Regional PSS programme for GIZ	Germany
**El Chammay,** Rabbih	Director of Mental Health, Ministry of Population Health, Lebanon	Lebanon
**Harmer**, Anne	Head of R2HC Programme at Elrha	UK
**Jones**, Cecilia Vaca	Executive Director, Bernard van Leer Foundation,	Ecuador
**Kemmer**, Danielle	Executive Director, International Alliance of Mental Health Research Funders	Canada
**Lukwata**, Hafisa	Focal point mental health, Ministry of Health, Uganda	Uganda
**Sridhar**, Priti	Mariwala Health Initiative	India
**Staglin**, Garen	Founder and Board Chairman, OneMind	USA
**Souza**, Renato	Chief, Mental Health and Substance Abuse, PAHO	Brazil
**Van der Waals**, Renet	Coordinator at Ministry of Foreign Affairs, The Netherlands	Netherlands
**Wolpert**, Miranda	Head of Mental Health Programme, Wellcome Trust	UK

**MHPSS-SET 2 Scientific and Practice Advisory Board (SPAB)**		
**Tol,** Wietse A. *Co-Chair*	University of Copenhagen & HealthRight International	Denmark
**Harrison,** Sarah *Co-Chair*	IASC Reference Group on MHPSS<br>IFRC PS Center	Denmark
**Annan**, Jeanie	International Rescue Committeee	USA
**Betancourt**, Theresa	Boston College	USA
**Bizouerne**, Cécile	Action Contre La Faim	France
**Bolton**, Paul	Johns Hopkins University/USAID	USA
**de Castro**, Elizabeth	University of Philippines	Philippines
**Eaton**, Julian	London School of Health and Tropical Hygiene & CBM	UK
**Engels**, Michelle	IFRC PS Center	Denmark
**Hijazi**, Zeinab	UNICEF	USA
**Horn**, Rebecca	Church of Sweden;<br>Queen Margaret University	UK
**Kiyanda**, Eugene	MRC Uganda	Uganda
**Kohrt**, Brandon	George Washington University	USA
**Koyiet**, Phiona	World Vision International	Kenya
**Onyango**, Patrick	REPSSI	Uganda /<br>South Africa
**Panter-Brick**, Catherine	Yale University	USA
**Pluess**, Michael	Queen Mary University	UK
**Rahman**, Atif	University of Liverpool	Pakistan
**Silove**, Derrick	University of New South Wales	Australia
**Tomlinson**, Mark	University of Stellenbosch	South Africa
**Uribe**, Jose Miguel	Pontificia Universidad Javeriana	Colombia
**Ventevogel**, Peter	UNHCR	Switzerland
**Weissbecker**, Inka	WHO	Switzerland
**Wessells,** Michael	Columbia University	USA

Appendix #2. Phase 1 Methodology, Generating Research Questions

Phase 1A Nominations of Expert Panel

All SPAB members were asked to nominate, via email or an online form, individuals to be invited to be part of the expert panel. We also invited the nominated individuals to nominate their colleagues through two rounds of outreach. We further invited leaders and co-leaders of the IASC Reference Group Technical Working Groups to participate in the study. A total of 374 members (including SPAB members) were nominated in Phase 1A; 227 (61%) completed survey #1 (generating research questions).

Table A1. Types of expert panel stakeholders who completed Survey #1 (n=227)

Type of Stakeholder	n	(%)
Researcher in LMIC	51	(22%)
Researcher in HIC	29	(13%)
Implementer in LMIC	89	(39%)
Implementer in HIC	8	(4%)
Policymaker in LMIC	21	(9%)
Policymaker in HIC	4	(2%)
Unknown	25	(11%)
**Total**	**227**	**(100%)**

Phase 1B: Humanitarian Agency-led Consultations

Led by MHPSS.net, we conducted a social media campaign on major platforms (e.g., Facebook, Twitter, WhatsApp) to invite MHPSS implementers, especially IASC MHPSS Technical Working Group members, to conduct "DIY Consultations" in their respective organisations. We conducted a webinar (13th October 2020) to provide information about the project and the DIY Consultation process to interested parties. The recording of the webinar (https://bit.ly/mhpss-set2DIYdemo) was made available, along with a "DIY Consultation Toolkit" (https://bit.ly/mhpss-set2DIYkit) to those who signed up to lead DIY Consultations in their organisations. Team leaders for DIY consultations were asked to submit the DIY Consultation Summary Form, available via an online survey (https://bit.ly/mhpss-set2diysummary), and a table format that could be submitted via e-mail. Each DIY Consultation could propose up to 10 questions.

Figure A1. Type of organizations in humanitarian agency-led consultations

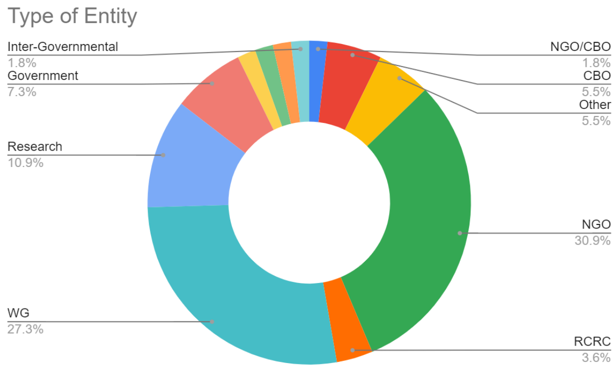

Phase 1C: Qualitative Study **Rationale**: The aim of the qualitative study was to ensure that voices from direct MHPSS implementers and those affected by humanitarian crises were heard to some extent, and could feed into the research priority setting process. We opted for qualitative interviews rather than surveys, as we expected that it would be easier for persons with lived experience to participate in interviews (given potential concerns with experience with surveys), and because – for MHPSS providers and policy makers – we were interested in a broader understanding of the context and narratives that shaped opinions on MHPSS research priorities.

Site selection and participants: We selected three sites, with the key rationale to ensure diversity in perspectives. We selected sites representing different types of humanitarian crises in different geographical settings: Uganda (post-conflict, hosting refugees), Lebanon (industrial disaster, hosting refugees), and Indonesia (disasters triggered by natural hazards). For each site, we hired an in-country team leader to recruit and conduct in-depth interviews (IDIs) and focus group discussions (FGDs) with local MHPSS service users (people with lived experience), implementers, and policymakers. The project manager (PTL) met virtually with each in-country team leader to train on the project methodology and data collection procedures. All team leaders were provided with the study instruments and detailed instructions on the process of submitting collected data.

Procedures: In-country team leaders recruited participants and were instructed to ensure participation of all three types of stakeholders (people with lived experience, implementers, and policymakers) and those with different backgrounds and experiences (gender, rural/urban, type of humanitarian crisis) as much as possible. Participants were recruited via flyers and personal communications with local networks of MHPSS programs and implementers. Semi-structured interview guides for IDIs and FGDs were developed by the Implementation Team and were translated and locally adapted by the in-country team leaders.

Participants were asked to engage in a discussion about what research questions should be focused on in the field of MHPSS over the next 10 years in order to strengthen MHPSS implementation in humanitarian crisis settings. Participants were asked to enumerate research questions on index cards, assign a priority-ranking to each question (high, moderate, or low), and subsequently complete a pile-sorting exercise to group research questions into overarching themes. During the pile-sorting exercise participants were asked to describe and provide rationale for the themes they identified. Finally, participants were asked to rank the identified themes in order of importance.

Additionally, IDIs/FGDs with service users were probed for both 'positive' and 'negative' perceptions that people may have about MHPSS programs in humanitarian settings, whereas IDI/FGDs with implementers probed for ways that research has improved or hindered MHPSS program implementation in humanitarian settings. Moreover, implementers were also asked to provide recommendations on how to reduce barriers to and strengthen the implementation of MHPSS programs in these settings.

Following data collection, in-country team leaders completed 'Summary Sheets' designed to synthesize data from each FGD and IDI. These summary sheets captured information on: (1) the research questions generated and their corresponding priority-rankings and rationales; (2) participant perceptions of MHPSS programs and research; and (3) interviewer observations throughout the data collection process, including notes about significant consistencies or inconsistencies in priority-ranking rationales provided by participants. Administrative

information such as site, stakeholder group, and method of data collection (IDI vs. FGD) was also recorded. Summary sheets containing only de-identified data were then provided to the US-based implementation team for final analysis

We obtained IRB approval from New York University (IRB#FY2020-4456). The sample sizes for the qualitative study participants are indicated in Table A2 below.

Data analysis: Questions generated from the qualitative study were combined along with questions generated from both the expert panel and the humanitarian agency-led consultations, yielding the consolidated list of 61 research questions categorized in 6 themes. Separate thematic analysis of the qualitative data on the positive and negative perceptions of MHPSS activities/programs was also undertaken. Trained research assistants consolidated participats' responses from the summary sheets into one document. In each question/topic, initial inductive themes were proposed with participant's responses categorized into the "best fitting" category, although some responses could fit in more than one category. An organizational scheme was created to re-organize categories into major themes and subthemes. The research team then read through the proposed themes, subthemes, and participant responses and revised them as necessary. In-country research coordinators commented on and revised the qualitative analysis, providing important on-the-ground perspectives.

Table A2. Number of IDIs and FGDs Participants in the Qualitative Study in Lebanon, Indonesia, and Uganda

	**Lebanon**	**Indonesia**	**Uganda**	**Total**
**IDIs**	**9**	**16**	**8**	**34**
People with lived experience	5	4	2	**11**
Implementers	3	10	6	**19**
Policy makers	1	2	1	**4**
**FGDs (# participants)**	**1 (7)**	**6 (57)**	**1 (4)**	**8 (68)**
People with lived experience	-	2 (10, 9)	1 (4)	**3 (23)**
Implementers	1 (7)	4 (10, 8, 10, 10)	-	**5 (45)**

Appendix #3. Prioritized Research Questions from 2011 Priority Setting Process

Rank	Category	Question	Significance	Answerability	Applicability	Equity	Ethics	Average
1	PA	1. What are the stressors faced by populations in humanitarian settings?	85.2	93.8	85.2	87.7	81.5	86.7
2	RI	70. What are appropriate methods to assess mental health and psychosocial needs of populations in humanitarian settings?	88.9	82.7	86.4	85.2	86.4	85.9
3	PA	11. How do affected populations themselves describe and perceive mental health and psychosocial problems in humanitarian settings?	88.9	86.4	80.2	86.4	87.7	85.9
4	RI	71. What are appropriate indicators to use when monitoring and evaluating the results of mental health and psychosocial support in humanitarian settings?	93.8	75.3	87.7	82.7	87.7	85.4
5	MI	14. How can we best adapt existing mental health and psychosocial interventions to different socio-cultural settings?	87.7	76.5	87.7	85.2	88.9	85.2
6	MI	16. What is the effectiveness of family-based interventions to prevent mental disorders and protect and promote psychosocial wellbeing and mental health among children and adolescent in humanitarian settings?	96.3	80.2	80.2	85.2	81.5	84.7
7	PA	7. What are the major protective factors (including individual [e.g. coping, hope] and contextual [justice mechanisms, religious practices]) for mental health and psychosocial problems in humanitarian settings?	90.1	77.8	84.0	82.7	87.7	84.5

Rank	Category	Question	Significance	Answerability	Applicability	Equity	Ethics	Average
8	MI	17. What is the effectiveness of school-based psychosocial and mental health interventions to prevent mental disorders and protect and promote psychosocial wellbeing and mental health among children and adolescent in humanitarian settings?	88.9	77.8	86.4	84.0	79.0	83.2
9	MC	47. To what extent do current mental health and psychosocial supports address locally perceived needs?	81.5	81.5	79.0	85.2	85.2	82.5
10	PA	2. Which are the most common mental health and psychosocial problems in the general population in humanitarian settings?	79.0	87.7	82.7	81.5	80.2	82.2
11	MC	62. What are the best ways to organize training and supervision of people delivering mental health and psychosocial support in humanitarian settings?	90.1	80.2	81.5	76.5	81.5	82.0
12	MC	42. What are the social and economic impacts of mental health and psychosocial support in humanitarian settings?	91.4	66.7	77.8	86.4	84.0	81.3
13	PA	13. How does the effectiveness of mental health and psychosocial support vary across specific populations groups (e.g. women, children, elderly, forced migrants, homeless children, people with disabilities, religious/ ethnic groups)?	87.7	65.4	77.8	88.9	86.4	81.2
14	PA	15. How can we best develop existing mental health and psychosocial interventions within different socio-cultural settings?	85.2	75.3	82.7	80.2	82.7	81.2

Rank	Category	Question	Significance	Answerability	Applicability	Equity	Ethics	Average
15	MI	36. What is the effectiveness of interventions aimed at decreasing the mental health and psychosocial burden of humanitarian staff?	79.0	81.5	86.4	74.1	81.5	80.5
16	MC	50. What are existing strengths and capacities (e.g. community leadership, indigenous expertise, religious/ spiritual structures) of communities in humanitarian settings?	84.0	74.1	81.5	79.0	82.7	80.3
17	MC	55. How can sustainability of mental health and psychosocial support in humanitarian settings best be maximized?	88.9	63.0	80.2	84.0	82.7	79.8
18	PA	3. What are the most common mental health and psychosocial problems in specific population groups (e.g. women, children, elderly, forced migrants, homeless children, religious/ ethnic groups) in humanitarian settings?	81.5	82.7	79.0	84.9	70.4	79.7
19	PA	5. What are the impacts of mental health and psychosocial problems for other outcomes in humanitarian settings (including economic, physical health, educational, and social outcomes)?	88.9	59.3	80.2	85.2	84.0	79.5
20	MI	33. What are effective methods to prevent mental health and psychosocial problems/ promote mental health and psychosocial wellbeing in humanitarian settings?	82.7	70.4	85.2	81.5	76.5	79.3

Appendix #4. Geographic Distribution of Panel Members' Countries of Experience

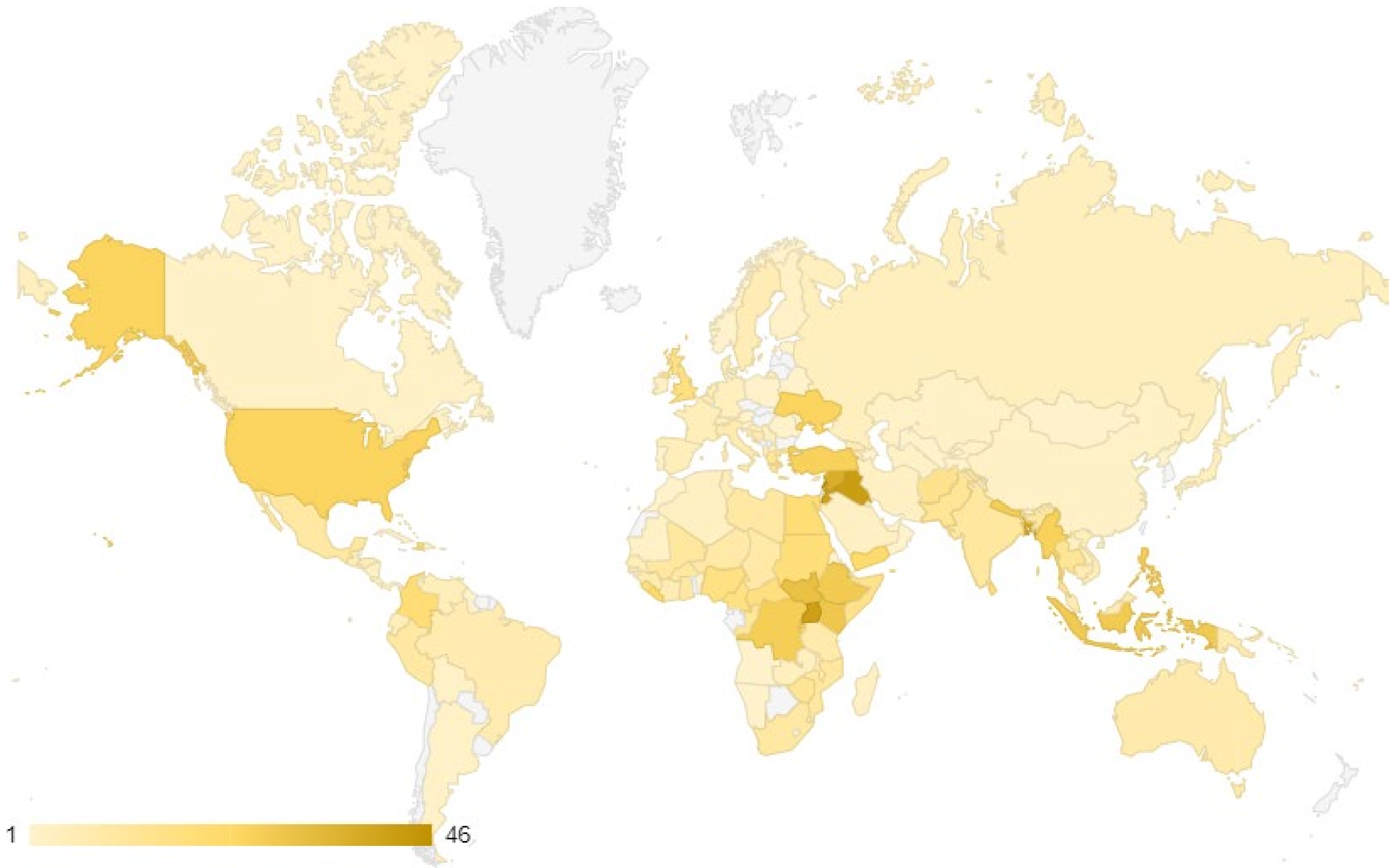

Appendix #5. Top 20 research questions by participant group

**Total Sample** (N=231)	**Researchers** (n=80)	**Implementers** (n=121)	**Policymakers** (n=15)
**10**	**27**	**22**	**61**
**11**	**11**	**10**	**5**
**22**	**10**	**11**	**11**
**27**	**4**	**4**	**15**
**4**	**29**	**27**	**20**
**1**	**1**	**15**	**23**
**61**	**3**	**1**	**47**
**3**	**30**	**61**	**56**
**9**	**17**	**9**	**4**
**25**	**31**	**20**	**9**
**30**	**61**	**25**	**22**
**19**	**22**	**19**	**27**
**15**	**9**	**45**	**28**
**5**	**25**	**3**	**30**
**29**	**19**	**47**	**52**
**47**	**21**	**6**	**57**
**6**	**14**	**28**	**58**
**20**	**5**	**5**	**21**
**24**	**6**	**30**	**24**
**45**	**24**	**29**	**25**

Research Questions

1	What are the most important MHPSS problems in humanitarian settings?*
3	How are the consequences of traumatic experiences and adversity, including childhood adversity, transmitted across generations?
4	How do mental health and psychosocial concerns influence social and economic functioning (e.g., economic outcomes, family functioning, social relations)?
5	What is the current understanding and gaps in knowledge about mental health and psychosocial support issues in humanitarian settings?
6	What are the major risk and protective factors of MHPSS issues in humanitarian settings?*
9	What are the correlates of resilience in humanitarian settings?
10	What is the impact of [MHPSS interventions] in humanitarian settings?*
11	What are the comparatively most optimal (e.g., effective, efficient, cost-effective, safe) MHPSS interventions/responses to address [issues] in humanitarian settings?*
14	What are the key working ingredients and mechanisms of change of MHPSS interventions?
15	What should be the minimum/essential set of MHPSS services in humanitarian settings?
17	How can we effectively design and/or implement trauma informed care?
19	How can we ensure effective participation of [key stakeholders] in MHPSS programs?*
20	What is the relationship between MHPSS programs and peacebuilding, and how can peacebuilding be effectively promoted in MHPSS programs?
21	How can we scale up effective MHPSS interventions in humanitarian settings?
22	How can we ensure the sustainability of MHPSS services in various settings and sectors?
23	What is the added value of, and how can we effectively integrate MHPSS services into primary health care in humanitarian settings?
24	What is the added value of integrating/mainstreaming MHPSS services into other sectors (e.g., education, WASH, social protection) in humanitarian settings?
25	How can we develop effective multi-sectoral, multi-layered interventions in humanitarian settings?
27	What are the appropriate methods to assess the outcomes and impact (short-term and long-term benefits) of [MHPSS interventions] and approaches?*
28	What are the appropriate methods to measure the quality of MHPSS interventions and approaches?
29	How can we develop and adapt tools that are culturally and cross-culturally valid?
30	How can we effectively develop MHPSS monitoring, evaluation, and research systems in humanitarian settings?*

31	How can we effectively translate research into practice and policy?
45	How can we better develop supervision models and strategies to address MHPSS needs in humanitarian settings?
47	How can we strengthen the MHPSS workforce (including community health and psychosocial workers, teachers, social workers, psychologists) in humanitarian settings?
52	How can we create infrastructure, governance, and network systems to promote effective MHPSS policies and responses?
56	What type of MHPSS is needed—and can be made available—during and following the Covid-19 pandemic or other disease outbreaks in humanitarian settings?
57	How can MHPSS interventions be adapted to address the Covid-19 pandemic?
58	What is the impact of digital technology, including social media, on individuals mental health and psychosocial wellbeing?
61	What are the effectiveness and best practices of remote/digital MHPSS interventions?

*Indicates research questions with drop-down options.
